# Humans seropositive for *Trypanosoma cruzi* co-infected with intestinal helminths have higher infectiousness, parasitaemia and Th2-type response in the Argentine Chaco

**DOI:** 10.1186/s13071-024-06401-7

**Published:** 2024-08-12

**Authors:** Gustavo Fabián Enriquez, Natalia Paula Macchiaverna, Graciela Garbossa, Luz Piedad Quebrada Palacio, Bárbara Leonor Ojeda, Jacqueline Bua, María Sol Gaspe, Rubén Cimino, Ricardo Esteban Gürtler, Miriam Postan, Marta Victoria Cardinal

**Affiliations:** 1 Universidad de Buenos Aires., Facultad de Ciencias Exactas y Naturales. Departamento de Ecología, Genética y Evolución. Laboratorio de Eco-Epidemiología., Buenos Aires, Argentina; 2 Instituto de Ecología, Genética y Evolución (IEGEBA), CONICET–Universidad de Buenos Aires, Buenos Aires, Argentina; 3 Laboratorio de Parasitología Clínica y Ambiental, Facultad de Ciencias Exactas y Naturales, Universidad de Buenos Aires, IQUIBICEN-CONICET-UBA), Instituto de Investigaciones en Salud Pública, Buenos Aires, Argentina; 4 Institute of Virology, Helmholtz Centre Munich, German Research Centre for Environmental Health, Neuherberg, Germany; 5 Instituto Nacional de Parasitología Dr. M. Fatala Chabén, Administración Nacional de Laboratorios e Institutos de Salud Dr. C.G. Malbrán, Buenos Aires, Argentina; 6 Instituto de Investigaciones de Enfermedades Tropicales (IIET). Consejo Nacional de Investigaciones Científicas y Técnicas (CONICET)–CCT Salta, Universidad Nacional de Salta, Sede Regional Orán, Salta, Argentina; 7 Facultad de Ciencias Naturales, Cátedra de Química Biológica, Universidad Nacional de Salta, Salta, Argentina; 8 Consejo Nacional de Investigaciones Científicas y Tecnológicas (CONICET), Buenos Aires, Argentina

**Keywords:** Gran Chaco, Neglected tropical diseases, *Trypanosoma cruzi*, Intestinal parasites, Co-infection, Heterogeneity, Immunomodulation

## Abstract

**Background:**

The Gran Chaco ecoregion is a well-known hotspot of several neglected tropical diseases (NTDs) including Chagas disease, soil-transmitted helminthiasis and multiparasitic infections. Interspecific interactions between parasite species can modify host susceptibility, pathogenesis and transmissibility through immunomodulation. Our objective was to test the association between human co-infection with intestinal parasites and host parasitaemia, infectiousness to the vector and immunological profiles in *Trypanosoma cruzi*-seropositive individuals residing in an endemic region of the Argentine Chaco.

**Methods:**

We conducted a cross-sectional serological survey for *T. cruzi* infection along with an intestinal parasite survey in two adjacent rural villages. Each participant was tested for *T. cruzi* and *Strongyloides stercoralis* infection by serodiagnosis, and by coprological tests for intestinal parasite detection. *Trypanosoma cruzi* bloodstream parasite load was determined by quantitative PCR (qPCR), host infectiousness by artificial xenodiagnosis and serum human cytokine levels by flow cytometry.

**Results:**

The seroprevalence for *T. cruzi* was 16.1% and for *S. stercoralis* 11.5% (*n* = 87). We found 25.3% of patients with *Enterobius vermicularis*. The most frequent protozoan parasites were *Blastocystis* spp. (39.1%), *Giardia lamblia* (6.9%) and *Cryptosporidium* spp. (3.4%). Multiparasitism occurred in 36.8% of the examined patients. Co-infection ranged from 6.9% to 8.1% for *T. cruzi*-seropositive humans simultaneously infected with at least one protozoan or helminth species, respectively. The relative odds of being positive by qPCR or xenodiagnosis (i.e. infectious) of 28 *T. cruzi*-seropositive patients was eight times higher in people co-infected with at least one helminth species than in patients with no such co-infection. *Trypanosoma cruzi* parasite load and host infectiousness were positively associated with helminth co-infection in a multiple regression analysis. Interferon-gamma (IFN-γ) response, measured in relation to interleukin (IL)-4 among humans infected with *T. cruzi* only, was 1.5-fold higher than for *T. cruzi*-seropositive patients co-infected with helminths. The median concentration of IL-4 was significantly higher in *T. cruzi*-seropositive patients with a positive qPCR test than in qPCR-negative patients.

**Conclusions:**

Our results show a high level of multiparasitism and suggest that co-infection with intestinal helminths increased *T. cruzi* parasitaemia and upregulated the Th2-type response in the study patients.

**Graphical Abstract:**

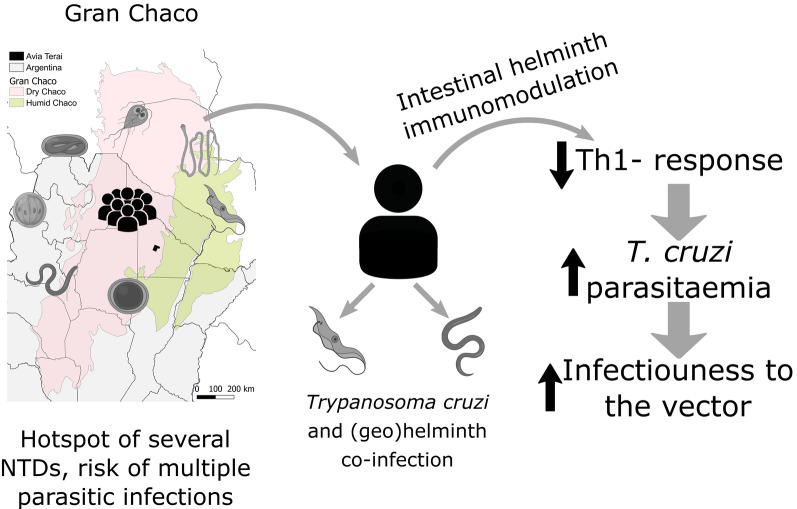

**Supplementary Information:**

The online version contains supplementary material available at 10.1186/s13071-024-06401-7.

## Background

Chagas disease, with the protozoan *Trypanosoma cruzi* as aetiological agent, is a neglected tropical disease (NTD) and remains a major public health problem in the American continent. It is estimated that 5–7 million people are infected and approximately 30% of them will be potentially affected by the disease [1]. Diagnosis of chronic human infection with *T. cruzi* for clinical purposes is based on the composite outcome of two serological tests with antigens that detect different antibodies against *T. cruzi* [enzyme-linked immunosorbent assays, (ELISA), indirect hemagglutination assay (IHA) or indirect immunofluorescence (IIF)] plus a third test if discordant results are obtained. However, the use of an ELISA or an immunochromatographic test is recommended for seroepidemiological surveys [2, 3]. Several regional intergovernmental initiatives coordinated intensified efforts to control the main triatomine species of public health relevance through the domestic application of pyrethroid insecticides [4]. However, the emergence of pyrethroid resistance in *T. infestans* and other vector species and associated control failures have jeopardized these achievements [5–7].

The Gran Chaco ecoregion is a hotspot of chronic poverty, Chagas disease and intestinal helminth infections, including soil-transmitted helminthiasis (STH) [8]. Soil-transmitted parasitic nematodes of humans include *Ascaris lumbricoides* (roundworms), *Trichuris trichiura* (whipworms), *Necator americanus* and *Ancylostoma duodenale* (hookworms) and *Strongyloides stercoralis*. Disease pathology caused by STH infections, mainly associated with heavier worm burdens, shares several features: diarrhoea and abdominal pain, anaemia and impaired growth and physical development [9, 10]. STHs, not considered major causes of global mortality [11], are the main cause of disability, with almost 2 million annual disability-adjusted life years (DALYs) worldwide [12]. Examination of stool samples by several methods has been recommended for diagnosis of STH infections [13]. However, because of the intermittent larval excretion during *S. stercoralis* chronic infection, serodiagnosis is more sensitive than faecal-based methods. Sensitivity of serological tests for *S. stercoralis* diagnosis ranged between 75% and 94%, whereas specificity ranged between 92% and 100%, depending on the study population, serologic methods (IIF or ELISA), antigen and reference method employed [14].

Other intestinal parasites co-circulate in the Gran Chaco ecoregion [15–21]. *Enterobius vermicularis* mainly affects children asymptomatically or with perianal pruritus as the main symptom. A strong positive correlation was found between *E. vermicularis* infection and appendicitis [22]. *Giardia* sp. and *Cryptosporidium* spp. cause moderate-to-severe diarrhoea and can also be accompanied by colic and nausea [23]. *Blastocystis* spp., one of the most frequent protozoa infecting humans, was associated with abdominal discomfort, anorexia, diarrhoea and flatulence [23, 24]. Other studies have suggested that *Blastocystis* is a non-pathogenic protozoan, as it is frequently seen in healthy individuals (e.g. [23]).

Multiparasitism (i.e. co-infection by more than one parasite species in the same host) is the rule in co-endemic areas for several parasitic diseases. Interspecific interactions between concomitant parasite species can modify host susceptibility to other parasites, their pathology and transmission risk [25, 26]. These features have favoured a major shift towards a multi-host and multi-parasite paradigm in research and control of infectious diseases [27, 28]. The main suggested mechanism of interaction between parasite species is the modulation of the immune response [29, 30]. In general, helminth infections mainly activate Th2 and Th9 immune response plus regulatory cytokines [31], polarizing the host immune response and reducing the ability to carry out an effective Th1-type response against intracellular parasites such as *T. cruzi*, *Toxoplasma gondii* and *Plasmodium* sp. [32]. Th17 has an important role in the control of *T. cruzi* infection [33, 34].

Several studies have provided evidence of immunomodulation by intestinal helminths and filarial worms in humans, especially in co-infections with *Plasmodium* sp. [35–37]. Immunomodulation was suggested as the underlying mechanism in *T. cruzi*-seropositive dogs co-infected with *Dirofilaria immitis*, which showed less intense heart inflammatory response than helminth-free dogs [38]. *Trypanosoma cruzi*-seropositive children with concomitant intestinal infections had high parasite loads, and malnutrition/growth retardation was up to four times higher than the national average in El Salvador [39]. The seroprevalence of *T. cruzi* and the detectability of bloodstream parasite DNA were significantly higher in Latin American immigrants co-infected with *S. stercoralis* than in those without the helminth [40, 41].

In vector-borne diseases, host infectiousness is the ability of an infected host to infect the vector; this can be measured directly by xenodiagnosis or indirectly by host parasitaemia. For *T. cruzi*, host infectiousness measured by xenodiagnosis is strongly overdispersed in humans and domestic or wild mammals [42–46]. These patterns involve ‘superspreaders’, defined as a small fraction of the host population infecting disproportionately more susceptible contacts than most infected hosts [47]. The bloodstream parasite load measured by quantitative PCR (qPCR) in humans and domestic or wild mammals naturally infected with *T. cruzi* was positively associated with host infectiousness and supported the 80–20 rule [48, 49]. Concomitant parasitic infections may in part account for the observed heterogeneous distribution of parasitaemia and infectiousness in mammalian populations infected with *T. cruzi*.

Here, we tested whether human co-infections with *T. cruzi* and intestinal parasites modified host parasitaemia, infectiousness to the vector and immunological profiles in an endemic rural area of the Argentine Chaco. Immunological profiles were measured through serum levels of Th1 (interferon-gamma, IFN-γ), Th2 (interleukin 4, IL-4), Th17 (IL-17A) and IL-10 cytokines. Our hypothesis is that intestinal helminth co-infection modulates the immune response towards a Th2-type response and thus increases host parasitaemia and infectiousness to the vector. No study apparently has addressed this research question in patients residing in an endemic region.

## Methods

### Study area

This study is part of a broader research project on the transmission and control of Chagas disease in the Argentine Chaco. This project, launched in 2015, encompassed a rural-to-urban gradient in Avia Terai municipality (26° 42′ S 60° 44′ W), Chaco province, north-eastern Argentina. The main features of the study area were described elsewhere [50]. The urban setting had 1409 inhabited dwellings arranged in a 10 × 10 block matrix as of 2016. Peri-urban settings included 575 inhabited dwellings. The rural setting comprised 308 inhabited houses as of 2015. The intervention program against *Triatoma infestans* included a municipality-wide baseline assessment of house infestation with triatomines, house spraying with pyrethroid insecticides and periodic searches to assess house re-infestation between November 2016 and November 2019 [7]. After the triatomine control program was initiated, a cross-sectional serosurvey of human infection with *T. cruzi* and treatment campaign was carried out between June 2016 and August 2017 in cooperation with personnel from the only local hospital (Dr. Ezequiel P. Morante).

## Study population

We conducted a cross-sectional serological survey for *T. cruzi* infection in which residents of two adjacent rural villages (Lote 9 and Lote 10) were invited to the nearest healthcare post or school for blood sample extraction. In parallel, intestinal parasite surveys were coupled with the *T. cruzi* serosurvey. A total of 87 individuals out of 415 residents voluntarily agreed to participate in both surveys. The exclusion criteria were: (i) being younger than 9 months of age, since maternal antibodies to *T. cruzi* may still be detectable; (ii) children younger than 18 years of age with no parent or guardian that provided written consent; and (iii) adults unable to give consent. In total, 28 *T. cruzi*-seropositive humans were included to evaluate host infectiousness, bloodstream parasite load and cytokines concentration profiles and its association with intestinal parasite co-infection. These included 9 out of 14 *T. cruzi*-seropositive humans from the serosurvey in Lote 9 and Lote 10 villages, and 19 *T. cruzi*-seropositive humans from other rural and urban areas in Avia Terai. The mean age of the 28 *T. cruzi*-seropositive humans was 27.9 years old (standard deviation, SD, 12.5) (Table 1), and approximately half of the patients were women. All humans seropositive for *T. cruzi* were in the chronic phase of the infection.

**Table 1:** Detection of patent parasitaemia through qPCR and xenodiagnosis tests of *T. cruzi*-seropositive humans co-infected with intestinal parasite species. Avia Terai, Chaco, 2016–2017

Infection status	No. patients	Mean age (SD)	No.*T. cruzi*-infected human positive by		Median *T. cruzi* parasite load (Q1–Q3)
			qPCR	Xenodiagnosis	
*T. cruzi*-infected humans	13	28.3 (11.39)	2	2	0.00 (0.00–0.00)
*T. cruzi*-helminths co-infected humans	5	32.0 (17.0)	3*	3*	0.33 (0.00–0.74)
*T. cruzi*-protozoa co-infected humans	5	28.0 (10.1)	0	0	0.00 (0.00–0.00)
*T. cruzi*-helminths-protozoa co-infected humans	5	19.4 (8.7)	2	2	0.00 (0.00–0.14)
Total	28	27.3 (12.2)	7	7	0.00 (0.00–0.00)

^*^ *P* = 0.03

Following national guidelines, all *T. cruzi*-seropositive people aged ≤ 18 years were referred to the local hospital for aetiological treatment against *T. cruzi*. Local physicians also considered eligible for aetiological treatment seropositive people between 18 and 65 years of age who specifically requested to be treated.

## Serosurvey

Up to 5 ml of blood were extracted by venipuncture from all participants aged more than 2 years. A retractile lancet was used for younger children and the maximum blood obtained was 0.5 ml. Blood samples were allowed to clot at room temperature for up to 3 h. Each serum was separated by centrifugation at 3000 rpm for 15 min and allocated in triplicate vials. Two aliquots were stored at −20 °C for serodiagnosis and one at −80 °C for cytokine quantification.

## Serodiagnosis of *Trypanosoma cruzi*

Each participant was tested by a recombinant antigen enzyme-linked immunosorbent assay (ELISA Rec V3.0, Wiener) and by an indirect hemagglutination assay (IHA) (Polychaco, Buenos Aires, Argentina) or by an in-house IHA tested at the national reference centre for *T. cruzi* diagnosis; Instituto Nacional de Parasitología Dr. Mario Fatala Chabén (ANLIS-Malbrán, Buenos Aires, Argentina). Each sample was tested in duplicate in the ELISA test as reported [51]. Discordant samples were tested by an indirect immunofluorescence (IIF) at the national reference centre for final outcome. An individual was considered seropositive when reactive to at least two assays. At the time of performing routine tests prior to Chagas aetiological treatment, a 2 ml aliquot of blood was mixed with an equal volume of Guanidine-EDTA Buffer (GEB) for molecular diagnosis and quantification of the bloodstream load of *T. cruzi*, and 3 ml of blood was mixed with heparin for artificial xenodiagnosis.

## Serodiagnosis of *Strongyloides* stercoralis

An in-house enzyme-linked immunosorbent assay was performed with the NIE antigen (NIE-ELISA) as described elsewhere [52]. Each sample was tested in duplicate. The cut-off was estimated by a standard curve using a positive serum control from *S. stercoralis*-infected humans. A serum sample from a non-infected human was used as a negative control. The cut-off was set at 120 units/mL, corresponding to a sensitivity and specificity of 75% and 95%, respectively [52]. Positive and negative control sera were run in each ELISA plate.

## Artificial xenodiagnosis and host infectiousness

Artificial xenodiagnostic tests were performed with heparinized blood from *T. cruzi*-seropositive humans. For each test, 20 fourth-instar nymphs of laboratory-reared *T. infestans* (kept unfed for at least 3 weeks) were offered 3 ml of heparinized blood from each patient using a blood-feeding device [53]. The elapsed time between blood extraction and the onset of feeding was < 5 min. Each triatomine was individually examined for *T. cruzi* infection by optical microscopy at 400 × magnification at 30 days and 60 days after exposure. Host infectiousness was calculated as the total number of triatomines infected with *T. cruzi* divided by the total number of triatomines exposed to the infected host and examined for infection at least once.

## Quantitative DNA amplification

All GEB samples were heated in boiling water for 10–15 min. Prior to DNA extraction, a bacterial commercial plasmid, pQE (Qiagen, Valencia, CA, USA), was added to a 400 μl GEB aliquot as an internal standard of DNA extraction, and DNA was extracted as reported [46]. Purified DNA was eluted in 200 μl of distilled water and used as template for qPCR amplification. An ABI 7500 thermocycler (Applied Biosystems, Carlsbad, CA, USA) was used to amplify a *T. cruzi* satellite DNA of 140 bp flanked by the Sat Fw and Sat Rv oligonucleotides, highly conserved in the parasite genome. Samples were run in duplicate. A standard curve reflecting a dynamic range of *T. cruzi* between 0.1 and 10^6^ parasites/ml was used for DNA quantification as described elsewhere [54]. DNA quantification was normalized according to the  Discrete Typing Units of *T. cruzi* [55]. We expressed parasite DNA concentration as equivalent amounts of parasite DNA per ml (Pe/ml). The cut-off value of this method was 0.14 Pe/ml [54]. Samples in which parasite DNA was not detected by qPCR were considered to have a zero parasite load per ml.

## Stool sampling

The procedure for stool sample collection (at least three independent samples over seven consecutive days) was explained verbally to all patients, who were provided with labelled containers with 3 ml sodium acetate–acetic acid–formalin (SAF). Three glass slides with a cellophane adhesive tape for Graham’s test were given to patients for detection of *E. vermicularis*. Two sets of printed instructions were used to describe the stool and adhesive tape test collection [56].

## Coprological examination and identification of intestinal parasites

Coprological examination was carried out by two concentration methods: a flotation test using a saturated sodium chloride solution (specific gravity 1.20 g/ml) and a modified sedimentation test employing washings with saline solution while avoiding the usual lipid extraction step [57]. We performed four independent observations for the sedimentation test (one of them iodine-stained), and one observation for the flotation technique with the addition of a drop of iodine. In both cases, all helminth eggs detected in each preparation were identified to genus by comparison with atlas photomicrographs [58]. For the detection of intestinal protozoa, a preparation was made for each faecal sample processed by the sedimentation protocol and adding a drop of iodine. The preparation was entirely examined by optical microscopy at 400 × to detect the presence of protozoa by comparison with atlas photomicrographs [58]. *Cryptosporidium* spp. oocysts were detected in stool smears by staining with a modified Ziehl–Neelsen stain [59]. Smears were examined by optical microscopy at 1000 × and oocysts recognized by the morphometric characteristics of fuchsia-stained spherical or ovoid elements (length, 4–6 μm). A patient was considered infected with a helminth species when an egg or larvae was detected by at least one method of enrichment (i.e. flotation or sedimentation), and/or infected with a protozoan when cysts or coccidian oocysts were observed. Infected patients were referred for aetiological treatment.

## Multiplex assay for cytokine quantification

Human cytokine levels were measured in frozen serum samples using a bead-based multiplex assay, LEGENDplex™ Human Th Cytokine Panel (BioLegend, Inc., USA). We measured the levels of four cytokines: IFN-γ, IL-4, IL-10 and IL-17A. All samples were assayed according to the manufacturer’s instructions on a FACSAria II flow cytometer (BD Immunocytometry Systems). The concentration of each cytokine was determined using a standard curve, provided with the LEGENDplex™ kit, generated in the same assay. Serum samples were tested in duplicate in 96-well plates. The FCS files generated on the flow cytometer were analysed using BioLegend’s LEGENDplex™ Data Analysis Software.

## Data analysis

Agresti–Coull binomial 95% confidence intervals (CI) were used for proportions [60]. The proportion of qPCR- and xenodiagnosis-positive humans co-infected or not with helminths (i.e. *E. vermicularis* and/or *S. stercoralis*) was analysed using Fisher’s exact test. A nominal significance level of 5% was considered. We used Kruskal–Wallis or Mann–Whitney tests to compare the median parasite load of *T. cruzi* and cytokine concentrations among groups.

We included the following parasite species to assess the relationship between parasite co-infections and host infectiousness and parasite load of *T. cruzi*-seropositive humans: *E. vermicularis* (number of humans infected, *n* = 8), *S. stercoralis* (*n* = 5), *G. lamblia* (*n* = 3), *Cryptosporidium* spp. (*n* = 1) and *Blastocystis* spp. (*n* = 8). These parasites exert detrimental health effects and modify the immune response of infected humans. We did not include other less frequent or non-pathogenic parasite species (e.g. *Hymenolepis nana*, *Entamoeba histolystica/dispar*, *Dientamoeba fragilis*). To reduce the number of independent variables, intestinal parasites were pooled in two groups on the basis of their phylogenetic relationship. One group included *E. vermicularis* and *S. stercoralis* (i.e. helminths; *n* = 10); the other group included the protozoa *G. lamblia*, *Cryptosporidium* spp. and *Blastocystis* spp. (*n* = 10), five of which co-occurred with at least a helminth species. *Trypanosoma cruzi*-seropositive humans not infected with any intestinal parasite (*n* = 13) functioned as a negative control group.

Multiple logistic regression analysis with robust standard errors, implemented in Stata (Stata 15, Stata Corp, College Station, Texas), was used to evaluate the association between *T. cruzi* infection-status and intestinal parasites infection categorized in two groups of parasites, that is, helminths and protozoa. The sample included the 87 humans tested in the serosurvey. The relationship between host infectiousness to the vector or parasite load (response variables) of *T. cruzi*-seropositive humans and selected predictors was tested through random-effects multiple logistic regression and linear regression with robust standard errors, respectively. The sample included 28 *T. cruzi*-seropositive humans. Because the triatomines used in xenodiagnosis were clustered on individual humans, observations are not independent. Two logistic regression models were tested for infectiousness in which the dependent variable was the infection status of each triatomine used in xenodiagnosis. The first model assessed whether host infectiousness was associated with bloodstream parasite load (a continuous variable) and included age of the host. The second model evaluated the relationship between host infectiousness and co-infection with at least one parasite from each group, taken as dichotomous variables: helminths (*E. vermicularis* and/or *S. stercoralis*), and protozoa (*Blastocystis* spp. and/or *Cryptosporidium* spp. and/or *G. lamblia*). The parasite load was log-transformed and a multiple linear regression with robust standard errors was performed with selected predictors. The age of the host (measured in months) was included in all regression analyses as a potential confounder. The interaction term between parasite groups was dropped from the final model for host infectiousness due to converging problems. The Wald test examined the hypothesis that all regression coefficients were 0. Multicollinearity was evaluated by the variance inflation factor (VIF); the mean VIF values (< 2) indicated the absence of significant collinearity.

The comparison of serum cytokine levels was carried out considering the following human infection-status groups: (i) humans not infected with any of the studied parasites (i.e. control group; *n* = 4); (ii) humans infected with *T. cruzi* only (*n* = 6); (iii) humans infected with helminths only (i.e. infected with *E. vermicularis* and/or *S. stercoralis*; *n* = 6); (iv) humans infected with protozoa only (i.e. *Blastocystis* spp. and/or *Cryptosporidium* spp. and/or *Giardia* sp., *n* = 8); (v) *T. cruzi*-seropositive humans co-infected with helminths (*n* = 4); (vi) *T. cruzi*-seropositive humans co-infected with protozoa (*n* = 5); and (vii) *T. cruzi*-seropositive humans co-infected with both helminths and protozoa (*n* = 5). The immune response was summarized into one variable “*t*” (Eq. 1) [61] to allow for quantitative comparisons between the different infection-status groups; “*t*” is defined as the relative concentration of IFN-γ, as a driver of Th1-type response, in relation to the concentration of IL-4, as a driver of Th2-type response, plus IFN-γ, that is, in the total cytokine pool [61]. Therefore, it measures whether the immune response is skewed towards a Th1 or Th2 profile, and takes values between 0 and 1. Similarly, the immune response can be estimated in relation to IL-10.

1 $$t=\frac{IFN-\gamma }{\left[IFN-\gamma \right]+[\text{IL}-4\text{ or IL}-10]}$$

## Results

### *Trypanosoma cruzi* and intestinal parasite infection

The seroprevalence of *T. cruzi* was 16.1% among the 87 survey participants. Overall, 69.0% (60 of 87) of the individuals were infected with at least one intestinal parasite: 54.0% of them with intestinal protozoa, and 34.5% with helminths. The only three intestinal helminth species found by coprological or serological tests were *E. vermicularis* (25.3%), *S. stercoralis* (11.5%) and *H. nana* (1.0%). The enteropathogenic protozoa found were *Blastocystis* spp. (39.1%), *G. lamblia* (6.9%) and *Cryptosporidium* spp. (3.4%). We also detected *Entamoeba hystolytica*/*dispar* (8.0%), but the species identification remained inconclusive. We found other non-pathogenic protozoa such as *Entamoeba coli* (11.5%), *Endolimax nana* (16.1%), *Iodamoeba bütschlii* (5.7%) and *Dientamoeba fragilis* (11.5%) (Table S1).

Of the 87 study individuals, 74.7% were infected with at least one intestinal parasite species and/or *T. cruzi*. The prevalence of multiparasitism was 36.8%. The prevalence of co-infection with *T. cruzi* and a helminth was 8.1%. The prevalence of *T. cruzi* and *S. stercoralis* co-infection was 2.3%, and 5.8% for co-infection with *T. cruzi* and *E. vermicularis.* The prevalence of co-infection with *T. cruzi* and protozoa was 6.9%; 4.6% with *Blastocystis* spp., and 1.2% with *G. lamblia*. We found no *T. cruzi*-infected patient co-infected with *Cryptosporidium* spp. and *H. nana*. Multiple logistic regression showed that the relative odds of being seropositive for *T. cruzi* was significantly associated with age of host (OR 1.04; 95% CI 1.01–1.07, *P* < 0.01) and was not significantly associated with infection with a protozoa (OR 0.57; 95% CI 0.22–1.51, *P* = 0.26), helminth (OR 2.8; 95% CI 0.80–9.76, *P* = 0.95) or with a helminth and protozoa co-infection (OR 5.45; 95% CI 0.43–69.07, *P* = 0.19) (Wald *χ*^2^ = 10.71; *P* = 0.01).

## Association between host infectiousness and parasitaemia in co-infections with intestinal helminths and *protozoa*

Of 28 *T. cruzi*-seropositive humans, 7 (25%, 95% CI 13–43) were positive by qPCR and xenodiagnosis with 100% concordance (Table 1). Among the 13 individuals seropositive for *T. cruzi* only, only 2 were qPCR-positive and xenodiagnosis-positive; 5 patients positive by qPCR and xenodiagnosis were co-infected with helminths, and 2 of these were also co-infected with protozoa. Therefore, having a detectable parasitaemia by qPCR or xenodiagnosis was significantly associated with a *T. cruzi*-helminth co-infection (Fisher’s exact test, *df *= 1, *P* = 0.03) (Table 1). The relative odds of having a positive test result by qPCR and xenodiagnosis was eight times higher in individuals co-infected with helminths than in those with no such co-infection (95% CI 1.35–47.41). Detectability of *T. cruzi* DNA by qPCR was also marginally significantly higher in patients co-infected with each one of the helminth species than in those with no co-infection. Three of five *S. stercoralis* co-infected patients (Fisher’s exact test, *df *= 1, *P* = 0.08; OR 7.13, 95% CI 1.04–48.58) and four of eight *E. vermicularis* co-infected ones were qPCR-positive (Fisher’s exact test, *df *= 1, *P* = 0.08; OR 5.67, 95% CI 1.00–32.26).

## *Trypanosoma cruzi *parasite load and infectiousness to *Triatoma infestans*

The median parasite load of *T. cruzi* was 0.00 Pe/ml in all infection-status groups except for the group co-infected with at least one helminth (*n* = 5, median parasite load = 0.33 Pe/ml, Q1–Q3 = 0.00–0.74), which was marginally significantly higher than patients infected with *T. cruzi* only (Kruskal–Wallis test, *df *= 1, *H* = 2.04, *P* = 0.06) (Table 1). Log-parasite load was positively associated with a helminth co-infection (Coef. = 0.98, 95% CI −0.005 to 1.971, *P* = 0.05), but not with host age or co-infection with a protozoan (Table 2; *P* ˃ *F* = 0.04) in a multiple linear regression.

**Table 2:** Parasite load and infectiousness to *T. infestans* of *T. cruzi*-seropositive humans according to potential risk factors adjusted for age of the host; Avia Terai, Chaco, 2016–2017

Potential risk factors	No. of patients	Parasite load		Infectiousness to the vector^a^	
		Coeff. (95% CI)	*P*	OR (95% CI)	*P*
Age of host in months	28	−0.002 (−0.004, 0.001)	0.070	0.99 (0.99, 1.01)	0.20
Only *T. cruzi* infection	13	0	−	1	−
*T. cruzi-*helminths co-infection	5	0.98 (−0.005, 1.971)	0.051	20.83 (1.37, 316.78)	0.03
*T. cruzi*-protozoa co-infection	5	−0.210 (−0.662, 0.243)	0.348	0.09 (0.01, 1.67)	0.11
*T. cruzi-*helminths-protozoa co-infection	5	−0.721 (−1.904, −0.462)	0.220	–	–

^a^The interaction term between parasite groups was dropped from the final model due to converging problems.

**Fig. 1:**
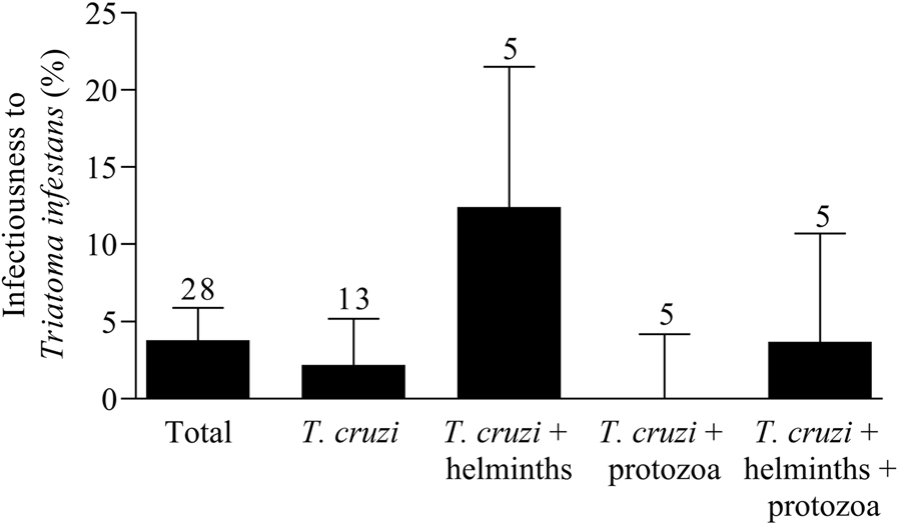
Mean infectiousness to *T. infestans* of *T. cruzi*-seropositive humans co-infected with intestinal parasite species. Avia Terai, Chaco, 2016–2017. The bars show mean host infectiousness of each group of individuals seropositive for *T. cruzi* and their co-infection status. The whiskers above each bar represent the 95% confidence interval, and the number of humans in each case is also indicated

In the xenodiagnostic tests, bug mortality and moulting rates at 30 days of post-exposure to heparinized blood from seropositive humans were 8% and 10%, respectively, indicating they were of acceptable quality though less than before [45]. The mean infectiousness to the vector was 3.8% (*n* = 477 triatomines, 95% CI 2.4–5.9). Infectiousness significantly increased with *T. cruzi* parasite load (OR 6.54, 95% CI 1.47–29.14, *P* = 0.01) in an age-adjusted logistic regression (Wald *χ*^2^ = 6.23, *P* = 0.04). The mean infectiousness of the five patients co-infected with at least one helminth only was 12.4% (95% CI 6.9–21.3), which was 5.6-fold higher than in individuals in the negative control group, that is, *T. cruzi*-seropositive patients not co-infected with any intestinal parasite (2.2%, 95% CI 0.9–5.0) (Fig. 1). When all patients co-infected with helminths were included, the between-group difference was reduced to 3.6-fold (8.0%; 95% CI 4.6–13.3). People co-infected with protozoa were the least infectious (0.0%, 95% CI 0.0–4.3) and those co-infected with helminths and protozoa had a mean infectiousness of 3.7% (95% CI 1.3–10.2). Infectiousness was positively associated with being co-infected with at least one helminth (OR 20.83, 95% CI 1.37–316.78, *P* = 0.03), but not with a protozoan in a multiple logistic regression adjusted for age (Table 2; (Wald *χ*^2^ = 5.21, *P* = 0.16).

## Cytokine profiles

**Fig. 2:**
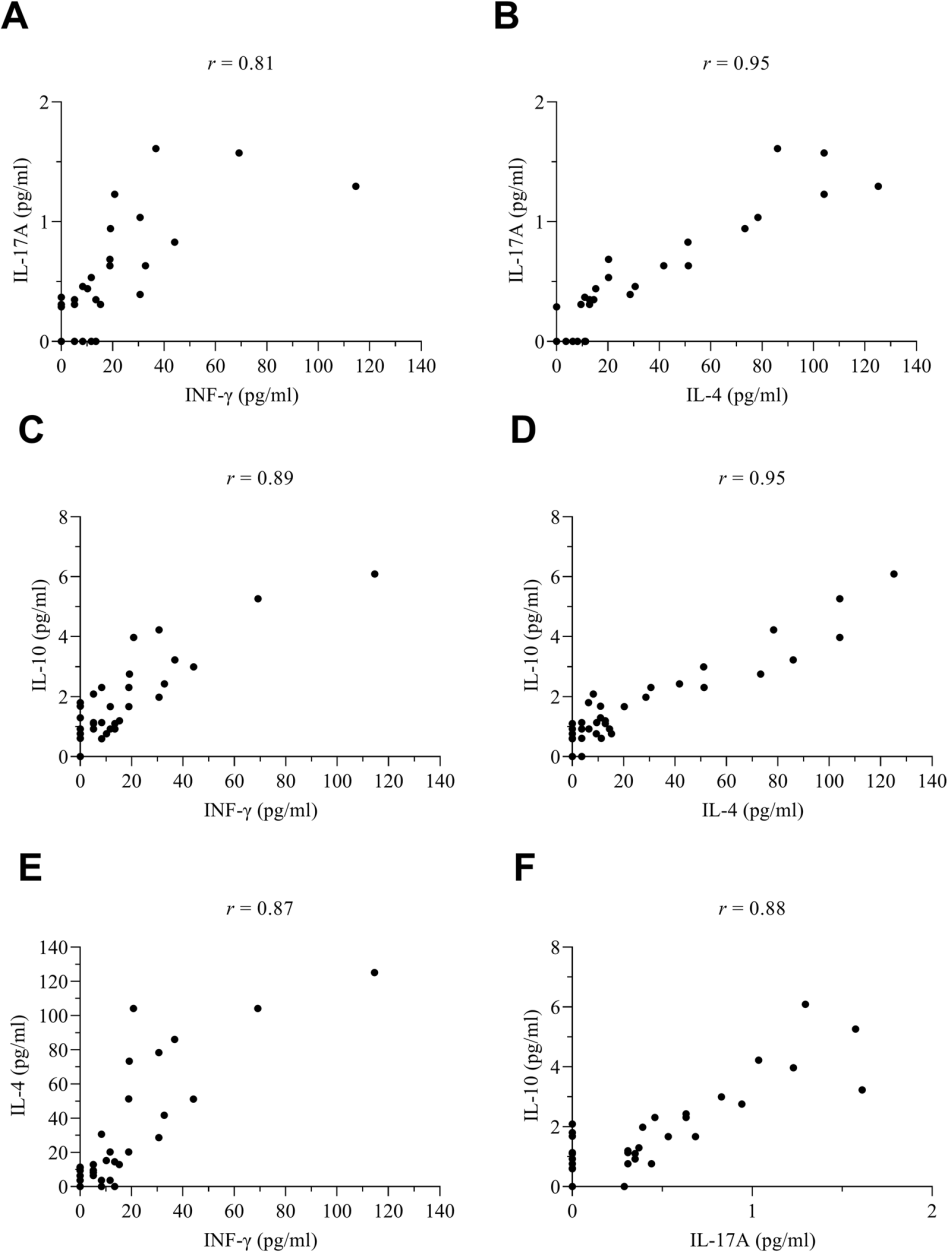
Pearson’s correlation coefficient between serum cytokine concentrations of humans. Avia Terai, Chaco, 2016–2017. **A** IL-17A versus IFN-γ, **B** IL-17A versus IL-4, **C** IL-10 versus IFN-γ, **D** IL-10 versus IL-4, **E** IL-4 versus IFN-γ, **F** IL-10 versus IL-17A

**Fig. 3:**
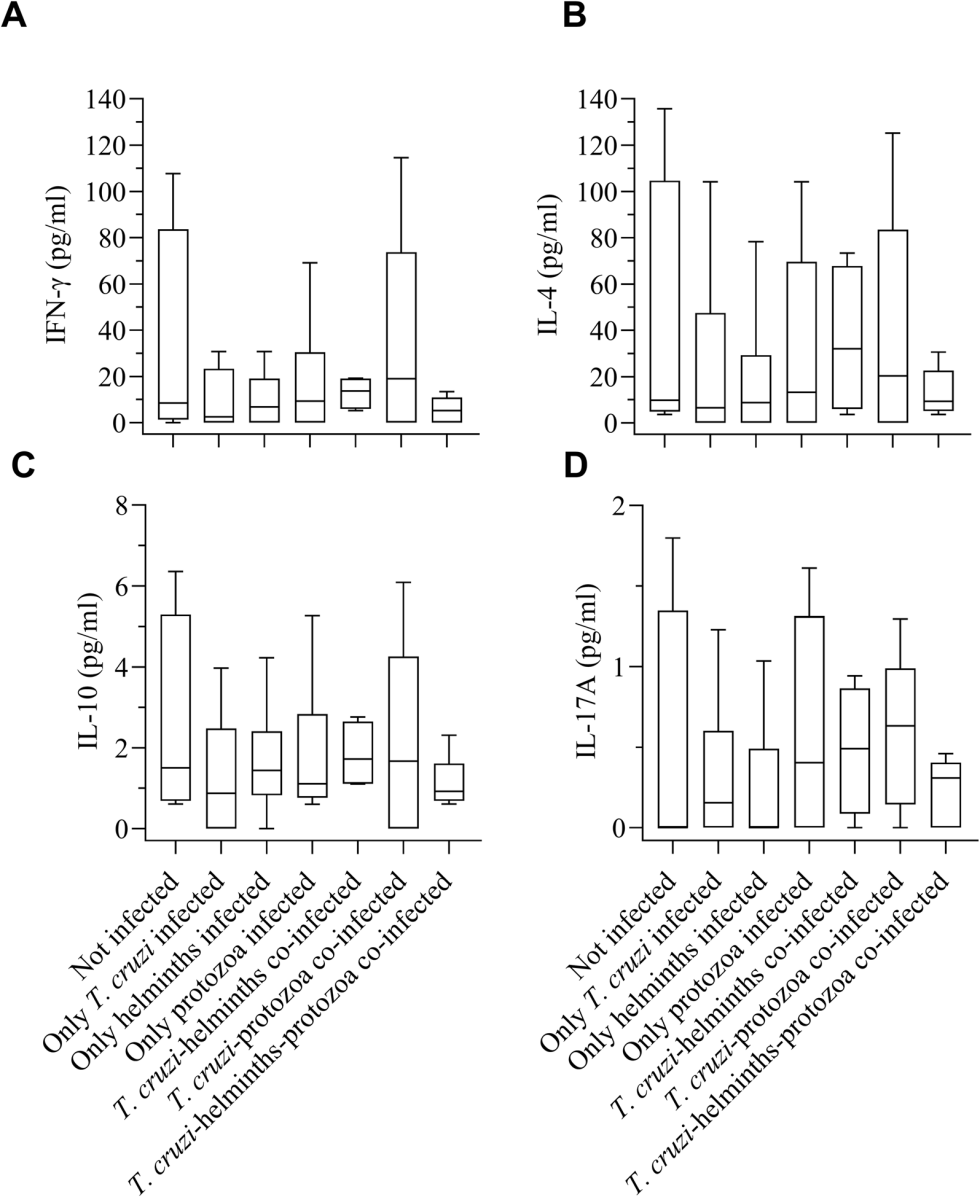
Serum cytokine concentrations of humans (*n* = 38) according to *Trypanosoma cruzi* and intestinal parasites infection. Avia Terai, Chaco, 2016–2017. **A** IFN-γ, **B** IL-4, **C** IL-10 and **D** IL-17A

The median cytokine concentration among the 38 tested patients was 1.14 picograms/milliliters (pg/ml) (Q1–Q3 = 0.77–2.31) for IL-10, 11.04 pg/ml (Q1–Q3 = 3.67–30.58) for IL-4, 0.31 pg/ml (Q1–Q3 = 0.00–0.63) for IL-17A and 8.34 pg/ml (Q1–Q3 = 0.00–18.93) for IFN-γ. There was a strong positive correlation among the four cytokine concentrations (range of Pearson’s correlation coefficients, *r*: 0.81–0.95, Fig. 2), with the weakest between IL-17A and IFN-γ. Median cytokine concentrations were not significantly different among infection-status groups (Kruskal–Wallis, range of *H* = 1.62–3.68, *df *= 6, *P* = 0.67–0.95) (Table S2). However, the median concentrations of IL-10 and IL-4 were the highest among *T. cruzi*-seropositive patients co-infected with at least one helminth, followed by those co-infected with protozoa (Fig. 3; Table S2). The opposite pattern was observed for IFN-γ and IL-17A: the median concentration was higher among *T. cruzi*-seropositive humans co-infected with protozoa followed by those co-infected with helminths. The median Th1-type response, measured by the relative concentrations of IFN-γ among humans infected with *T. cruzi* only, was the lowest among human infection-status groups when it was relativized by IL-10 (range of *t* = 0.41–0.92), and 1.5-fold higher than for *T. cruzi*-seropositive patients co-infected with helminths when it was relativized by IL-4 (Table S2).

Higher median cytokine concentration values were observed in *T. cruzi*- positive patients by qPCR (*n* = 5) compared with those found in *T. cruzi*-negative patients by qPCR (*n* = 15), regardless of co-infection status with intestinal parasites. Significantly higher IL-4 concentrations occurred in qPCR-positive patients compared with qPCR-negative patients (30.58 pg/ml versus 6.52 pg/ml, Mann–Whitney *U*-test, *Z* = −2.06, *P* = 0.04), while there were marginally significant differences for IFN-γ (18.93 pg/ml versus 5.20 pg/ml, Mann–Whitney *U*-test, *Z* = −1.83, *P* = 0.07) and IL-17A levels (0.46 pg/ml versus 0.29 pg/ml, Mann–Whitney *U*-test, *Z* = −1.74, *P* = 0.08). Furthermore there was no significant differences for IL-10 concentrations (Mann–Whitney *U*-test, *Z* = −1.67, *P* = 0.10) (Table 3).

**Table 3:** Cytokine concentrations according to qPCR results of 20 *Trypanosoma cruzi*-seropositive humans; Avia Terai, 2016–2017.

*T. cruzi*-qPCR results	No. of patients	Median cytokine concentration (Q1–Q3)			
		IL-4 (pg/ml)*	IL-10 (pg/ml)	INF-γ (pg/ml)	IL-17A (pg/ml)
Positive	5	30.58 (28.70–51.31)	2.31 (1.98–2.31)	18.93 (13.48–19.18)	0.46 (0.39–0.63)
Negative	15	6.52 (0.00–0.29)	0.92 (0.00–1.67)	5.20 (0.00–18.93)	0.29 (0.00–0.63)

^*^P < 0.05

## Discussion

Identifying factors and mechanisms that lead to overdispersed bloodstream parasite load in *T. cruzi*-infected host may provide a better understanding of vector-borne transmission dynamics and novel targeted control strategies. In this field study in the Argentine Chaco, we recorded nearly 75% of the study population infected with at least one intestinal parasite species and/or *T. cruzi*. As hypothesized, we found a positive association between the bloodstream parasite load of *T. cruzi* and co-infection with *S. stercoralis* and/or *E. vermicularis*. Infectiousness was higher in patients co-infected with *T. cruzi* and helminths, particularly when the co-infection excluded intestinal protozoa. Furthermore, *T. cruzi*-infected humans co-infected with helminths showed the highest Th2 immune response, suggesting it may exert a potential modulatory effect on *T. cruzi* parasitaemia.

Our study population resided in rural settings of northern Argentina, where the transmission of infectious diseases is strongly influenced by socio-economic and environmental factors characteristic of this ecoregion. In rural areas across Avia Terai municipality, other studies reported a high prevalence of house infestation with *T. infestans* (46%) and of houses harbouring *T. cruzi*-infected vectors (14%) [50, 62]. Rural households had characteristics suitable for transmission of intestinal parasites, such as lack of sanitary structures and access to safe drinking water, overcrowding and several unrestrained domestic animals that enhance the transmission of zoonotic intestinal parasites [63]. As NTDs and other infectious diseases share similar risk factors and there are potential interspecific interactions among parasite species, a multi-disease integrated approach may be necessary and cost-effective in regions with high co-endemicity [64, 65], as in the Gran Chaco.

Although we performed standard diagnostic methods that allow for the identification of several parasitic infections, the serological assay employed for *S. stercoralis* diagnosis (NIE-ELISA) has some limitations. In the first place, it is less reliable for acute infections or immunocompromised patients; cross-reactivity with other nematode infections may occur, and it cannot distinguish current from past infections. However, there are some points that suggest that seropositive patients were having a chronic *S. stercoralis* infection: (i) NIE-ELISA was among the first methods that revealed a seroreversion among five serological tests when infected patients underwent antihelminthic treatment [66]; (ii) infection with *S. stercoralis* could persist for years in the host because of autoinfection [67, 68]; and (iii) the study area is endemic for *S. stercoralis* and there has been no geohelminth management program to date.

In Avia Terai, the frequency of co-infections including *T. cruzi* and *S. stercoralis* (2%) was lower than elsewhere in northern Argentina (5%) [69]. We did not find a statistically significant association between these infections, nor did Fleitas et al. [69]. However, among Latin American expatriates, *T. cruzi* infection was more frequent in humans seropositive to *S. stercoralis* [41], and such co-infection was positively associated with eosinophilia [70]. The apparent lack of an association between human infection with *T. cruzi* and *S. stercoralis* or other intestinal parasites in co-endemic regions may perhaps be related to the inherent limitations of cross-sectional surveys for evaluating interspecific interactions between parasites [71, 72].

Half of the patients with *T. cruzi* infection were concomitantly infected with a pathogenic intestinal parasite. Patients co-infected with *T. cruzi* and *S*. *stercoralis,* or with *T. cruzi* and *E. vermicularis*, were more likely to harbour a detectable bloodstream parasite load by qPCR. This pattern agrees with previous results found in humans co-infected *T. cruzi* and *S. strongyloides* [40, 73], and here we expand it for humans co-infected with *T. cruzi* and *E. vermicularis*. Experimental animal models revealed an association between co-infection with intestinal helminths and *Leishmania* pathology or parasitaemia [74, 75]. Parasitaemia, qualitatively determined by blood culture, was higher in Golden lion tamarins naturally infected with *T. cruzi* and Trichostrongyloidae [76]. Similarly, a *T. cruzi*-helminth co-infection in dogs was associated with increased host parasitaemia and infectiousness [77].

Several studies have provided strong evidence that *S. stercoralis* may modulate disease severity in tuberculosis and was positively associated with higher bacterial burden [78–81]. Further, the low pathogenicity of *E. vermicularis* suggests it may have a low capacity to modulate the immune response of the infected host, and consequently, it would not affect the establishment or intensity of infection with other parasites. However, patients co-infected with *Plasmodium* malaria and *E. vermicularis* had higher parasitaemia and an increased risk of anaemia [82], supporting the hypothesis that helminth infections usually favour high microparasite loads. Accordingly, our results on *T. cruzi*-seropositive humans co-infected with two intestinal helminths suggest they may exert positive effects on host parasitaemia and infectiousness. If parasitaemia could be better controlled after deworming, this strategy may bring health benefits at the individual host and population levels by reducing the number and intensity of infectious hosts.

Co-infection with protozoa may also modulate the observed immune response. For example, *Giardia* spp. and *Cryptosporidium* spp. may affect the efficacy of vaccines against viral pathogens [83]. However, our results show that *T. cruzi* bloodstream parasite load and host infectiousness was not associated with *Giardia* spp. and/or *Cryptosporidium* spp. infection. This pattern was also reported for dogs co-infected with *T. cruzi* and *Cryptosporidium* spp. and/or *Giardia* spp. in the Argentine Chaco [77].

Patients co-infected with *S. stercoralis* and *Mycobacterium tuberculosis* had higher levels of Th2-type anti-inflammatory and regulatory cytokines (IL-4, IL-9, IL-10, TGF) and lower pro-inflammatory cytokine levels (IFN-γ, TNF- α, IL-2, IL-17) than patients infected with the mycobacteria only [78, 80]. Elevated plasma levels of IL-1β in association with anaemia, pyrexia and exacerbated parasitaemia occurred in patients co-infected with *Plasmodium falciparum* and *E. vermicularis* or multiple helminth species [82]. We found moderately higher concentrations of regulatory (IL-4 and IL-10) and pro-inflammatory (IFN-γ and IL-17A) cytokines in *T. cruzi*-seropositive humans co-infected with helminths or protozoa, respectively, compared with humans seropositive for *T. cruzi* only. Furthermore, IFN-γ response (measured in relation to IL-4) was lower for *T. cruzi*-seropositive humans co-infected with helminths than in patients infected with *T. cruzi* only. Altogether, we speculate that *S. stercoralis* and *E. vermicularis* infections exert an immunomodulatory phenomenon towards a Th2-type response in *T. cruzi*-infected patients inducing detrimental effects on the host’s ability to control parasitaemia with *T. cruzi*. A limitation of our study is the low number of serum samples evaluated for each infection/co-infection group; larger sample sizes are required to confirm the tendency found in serum cytokine levels.

Next, we analysed cytokine levels on the basis of the results of PCR and found that *T. cruzi*-infected patients with a positive qPCR had nearly five-fold higher levels of IL-4, a cytokine associated with Th2-type immune response respect to qPCR-negative patients. Th2-type IL-4 and IL-10 cytokines were crucial for downregulation of the pro-inflammatory response in *T. cruzi* infection [84, 85]. In experimental mouse models, increases of IL-4 were associated with increases of *T. cruzi* parasitaemia [86]; IL-4 depleted mice presented lower parasitaemia, associated with an increase of IFN-γ [87]. However, more research is required to determine whether the increase in IL-4 has a positive effect on the detection of patent parasitaemia in humans infected with *T. cruzi*, as well as to determine whether the concentration of IL-4 is due exclusively to the balance between the anti- and pro-inflammatory immune responses against *T. cruzi* infection or whether it is further exacerbated by co-infections with other parasite species. Our results support this last hypothesis, since the lowest levels of IFN-γ were found in relation to IL-4 in *T. cruzi-*seropositive patients co-infected with helminths. Interestingly, our study also recorded relatively elevated levels of IFN-γ and IL-17A in patients with patent parasitaemia by qPCR.

Amongst the strengths of our study are the collection of serial stool samples and the use of two concentration methods for parasitological diagnosis, use of differential staining to confirm the presence of *Cryptosporidium* spp. and use of ELISA for *S. stercoralis* diagnosis. However, we did not evaluate infection by virus or parasitic species with other locations in the human body, which may also influence host immune response affecting *T. cruzi* infection. One of the main limitations of this study is the low number of co-infected patients included. Despite the limited sample size, the difference in infectiousness and parasite load between infection-status groups was large enough to detect significant differences. By contrast, sample sizes were not sufficient to detect significant differences in serum cytokine levels. The stability of frozen cytokines, which is affected after 4 years of storage even at −80 °C [88], and differences in the balance of Th1/Th2/Th17/Treg adaptive immune responses between phase of the *T. cruzi* infection, that is, acute or chronic phase, asymptomatic patients and patients with the cardiac form of chronic Chagas disease [33, 34, 89], could also have added to the low discriminating power of the comparative analyses performed. The concentrations of the cytokines found in this study were close to or slightly below those found in other studies with parasitic infection, including patients with *T. cruzi* infection [90–93]. Imperfect detection in the qualitative and quantitative diagnostic methods can influence the results of analyses [94], especially in low concentrations, as it is the case of *T. cruzi* parasitaemia. However, the 100% of concordance between qPCR and xenodiagnosis test results suggest that the proportion of patients infected with *T. cruzi* who had detectable parasitaemia by these methods may be the true proportion of individuals with parasitaemia in the sample.

## Conclusions

Our study revealed high levels of multiparasitism in the Argentine Chaco. The prevalence of intestinal helminths was high to moderate, mostly represented by *E. vermicularis* and *S. stercoralis*. *Trypanosoma cruzi*-seropositive patients co-infected with helminths displayed substantially higher parasitaemia levels and host infectiousness; this may have important implications for the epidemiology and control of vector-borne transmission of *T. cruzi*. Heterogeneities in infectiousness, vector-host contact rates and host susceptibility to infection increase the basic reproduction number of pathogens [49]. Identifying the small fraction of superspreaders would allow for the design of intervention strategies targeting the population subgroups with a higher risk of infection or with a greater potential contribution to transmission. Experimental removal of helminth infections in *T. cruzi*-seropositive patients may confirm the effect of co-infections on *T. cruzi* parasitaemia and infectiousness to the vector and provide more evidence on the role of immunomodulation as an underlying mechanism.

### Supplementary Information

**Additional file 1: Table S1.** Frequency and prevalence of intestinal parasites found in humans from Avia Terai, Chaco, 2016–2017.

**Additional file 2: Table S2.** Cytokine concentration profiles of the study human population (*n* = 38) according to infection status groups. Avia Terai, Chaco, 2016–2017.

**Additional file 3: **Dataset. Results per individual (Database).

## Data Availability

The datasets supporting the conclusions of this article are included in the article (and its additional files).

## Abbreviations

ELISA: Enzyme-linked immunosorbent assays
GEB: Guanidine–EDTA blood samples
IAC: Internal amplification control DNA
IHA: Indirect hemagglutination assay
IIF: Indirect immunofluorescence
kDNA-PCR, SAT DNA-PCR: PCR amplification of the minicircle of kinetoplast DNA and satellite DNA, respectively
NTD: Neglected tropical disease
Pe: Parasite equivalents
qPCR: Quantitative PCR
SAF: acetate–acetic acid–formalin
STH: Soil-transmitted helminthiasis
Th: T helper type immune response
