# Supplementary material for: Humans seropositive for Trypanosoma cruzi co-infected with intestinal helminths have higher infectiousness, parasitaemia and Th2-type response in the Argentine Chaco
Source: Parasit Vectors. 2024 Aug 12;17:340. doi: 10.1186/s13071-024-06401-7 (PMC11320973; doi:10.1186/s13071-024-06401-7)
Supplement: Supplementary file 1 — Additional file 1: Table S1. Frequency and prevalence of intestinal parasites found in humans from Avia Terai, Chaco, 2016–2017. [file 13071_2024_6401_MOESM1_ESM.docx]

| Intestinal parasite species | No. tested samples | No. positive samples | Prevalence % (95% CI) |
| --- | --- | --- | --- |
| Helminth species |  |  |  |
| *Enterobius vermicularis* | 87 | 22 | 25.3 (17.3-35.4) |
| *Strongyloides stercoralis* | 87 | 10 | 11.5 (6.2-20.1) |
| *Hymenolepis nana* | 87 | 1 | 1.0 (0.2-6.2) |
| Protozoan species |  |  |  |
| *Blastocystis hominis* | 87 | 34 | 39.1 (29.5-49.6) |
| *Giardia lamblia* | 87 | 6 | 6.9 (2.9-14-5) |
| *Cryptosporidium* sp. | 87 | 3 | 3.4 (0.8-10.1) |
| *Entamoeba histolytica/dispar* | 87 | 7 | 8.0 (3.7-15.9) |
| *Entamoeba coli* | 87 | 10 | 11.5 (6.2-20.1) |
| *Endolimax nana* | 87 | 14 | 16.1 (9.7-25.3) |
| *Iodamoeba bütschlii* | 87 | 5 | 5.7 (2.2-13.1) |
| *Dientamoeba fragilis* | 87 | 10 | 11.5 (6.2-20.1) |

Table S1. Frequency and prevalence of intestinal parasites found in humans from Avia Terai, Chaco, 2016-2017.
