# Supplementary material for: Humans seropositive for Trypanosoma cruzi co-infected with intestinal helminths have higher infectiousness, parasitaemia and Th2-type response in the Argentine Chaco
Source: Parasit Vectors. 2024 Aug 12;17:340. doi: 10.1186/s13071-024-06401-7 (PMC11320973; doi:10.1186/s13071-024-06401-7)
Supplement: Supplementary file 2 — Additional file 2: Table S2. Cytokine concentration profiles of the study human population (n = 38) according to infection status groups. Avia Terai, Chaco, 2016–2017. [file 13071_2024_6401_MOESM2_ESM.docx]

Table S2. Cytokine concentration profiles of the study human population (n = 38) according to infection status groups. Avia Terai, Chaco, 2016-2017.

|  |  |  | Circulating cytokine profiles: Median (Q1-Q3) | | | |  | Median *t** | |
| --- | --- | --- | --- | --- | --- | --- | --- | --- | --- |
| Human infection status | No. patients | Mean age (standard deviation) | IL-10 (pg/ml) | IL-4 (pg/ml) | IL-17A (pg/ml) | IFN-γ (pg/ml) |  | IL-10 | IL-4 |
| Not infected | 4 | 28.8 (11.8) | 1.51 (0.61-2.09) | 9.77 (3.67-11.34) | 0.00 (0.00-0.00) | 8.46 (0.00-11.71) |  | 0.82 | 0.42 |
| Only *T. cruzi* infected | 6 | 27.2 (7.7) | 0.88 (0.00-1.98) | 6.60 (0.00-28.70) | 0.15 (0.00-0.39) | 2.60 (0.00-20.84) |  | 0.41 | 0.43 |
| Only helminths infected | 6 | 21.8 (17.9) | 1.44 (1.10-1.80) | 8.68 (0.00-12.86) | 0.00 (0.00-0.31) | 6.74 (0.00-15.25) |  | 0.44 | 0.39 |
| Only protozoa infected | 8 | 24.4 (12.6) | 1.11 (0.77-1.67) | 13.16 (0.00-20.29) | 0.41 (0.00-0.53) | 9.28 (0.00-11.71) |  | 0.9 | 0.40 |
| *T. cruzi-*helminths co-infected | 4 | 26.0 (12.0) | 1.73 (1.10-2.31) | 32.08 (3.67-51.31) | 0.49 (0.00-0.63) | 13.64 (5.20-18.93) |  | 0.88 | 0.28 |
| *T. cruzi-*protozoa co-infected | 5 | 31.4 (13.6) | 1.67 (0.00-2.43) | 20.29 (0.00-41.76) | 0.63 (0.29-0.69) | 18.93 (0.00-32.76) |  | 0.92 | 0.48 |
| *T. cruzi*-helminths-protozoa co-infected | 5 | 19.4 (8.7) | 0.92 (0.77-0.92) | 9.36 (6.52-14.54) | 0.31 (0.00-0.35) | 5.20 (0.00-8.34) |  | 0.78 | 0.21 |
| Kruskal Wallis H-test, P (H) | - | 0.58 (4.75) | 0.86 (2.55) | 0.95 (1.62) | 0.67 (3.68) | 0.88 (2.29) |  | - | - |

*Relative concentration of IFN-γ, as a driver Th1-type response, relative to the concentration of IL-10 or IL-4, as a driver Th2-type response, plus IFN-γ, i.e. in the total cytokine pool (Graham 2001).
